# TRCMGene: A two-step referential compression method for the efficient storage of genetic data

**DOI:** 10.1371/journal.pone.0206521

**Published:** 2018-11-05

**Authors:** You Tang, Min Li, Jing Sun, Tao Zhang, Jicheng Zhang, Ping Zheng

**Affiliations:** 1 Electrical and Information Engineering College, JiLin Agricultural Science and Technology University, Jilin, China; 2 College of Electrical and Information, Northeast Agricultural University, Harbin, China; 3 College of Life Science and Agriculture, Qiqihar University, Qiqihar, China; University of Adelaide School of Medicine, AUSTRALIA

## Abstract

**Background:**

The massive quantities of genetic data generated by high-throughput sequencing pose challenges to data storage, transmission and analyses. These problems are effectively solved through data compression, in which the size of data storage is reduced and the speed of data transmission is improved. Several options are available for compressing and storing genetic data. However, most of these options either do not provide sufficient compression rates or require a considerable length of time for decompression and loading.

**Results:**

Here, we propose TRCMGene, a lossless genetic data compression method that uses a referential compression scheme. The novel concept of two-step compression method, which builds an index structure using *K*-means and *k*-nearest neighbours, is introduced to TRCMGene. Evaluation with several real datasets revealed that the compression factor of TRCMGene ranges from 9 to 21. TRCMGene presents a good balance between compression factor and reading time. On average, the reading time of compressed data is 60% of that of uncompressed data. Thus, TRCMGene not only saves disc space but also saves file access time and speeds up data loading. These effects collectively improve genetic data storage and transmission in the current hardware environment and render system upgrades unnecessary. TRCMGene, user manual and demos could be accessed freely from https://github.com/tangyou79/TRCM. The data mentioned in this manuscript could be downloaded from: https://github.com/tangyou79/TRCM/wiki.

## Introduction

The advent of next-generation sequencing (NGS) techniques has enabled the rapid generation of an overwhelming and ever-growing amount of information [1]. Massive amounts of genetic data may exert intense stress on existing hardware environments. The computational concerns introduced by genetic data are related to the central processing unit (CPU) time required for data processing, storage and transmission[2]. In fact, storage systems are the real bottleneck in the processing of NGS data[1][3].

Compression involves storing genetic data to minimise storage cost and maximise computational and transferring efficiency[4]. Several options are available for compressing and storing genetic data. One possible solution is the use of general-purpose compression software, such as zip and GZip [5]. However, such compression software is not specifically designed for genetic data storage and analysis. Thus, these programs provide low compression rates, and decompression is always needed before data could be accessed. Other solutions, such as PLINK[6] and PBAT[7], have been proposed. These programs are free whole-genome association analysis toolsets introduced in binary PED formats, the most well-known compression format used in genome-wide association studies[8,9]. Nevertheless, its compression rate remains insufficient, and the compressed datasets of sequencing data could still occupy several gigabytes of disc space. In recent years, sophisticated compression techniques designed specifically for sequencing data have been proposed[10]. Many of these techniques are based on a referential compression schemes[11], such as DNAzip[12]. Referential compression approaches take advantage of shared information and store the relatively small differences between sequences and the reference sequence [11,13–15]. The compression rate of this scheme highly depends on the similarity between the reference sequence and to-be-compressed sequences[16].

Although useful, referential compression approaches suffer from several drawbacks, as follows: Firstly, a large overhead is needed for storing the reference sequence. Secondly, no given reference sequence may be available for compression[17]. Thirdly, poor similarities between the reference and to-be-compressed sequences may result in a low compression rate. A possible solution to these drawbacks is to dynamically select several particular sequences (called core sequences) from the pre-compression file by using a clustering algorithm[18]. These core sequences play the same role as the reference sequence in the compression process. Compression is performed by storing the differences between the to-be-compressed sequences and core sequences instead of those between the to-be-compressed sequences and reference sequences[19]. In the present study, we refer to this approach as the one-step referential compression method (ORCM). After the initial compression tests, some satisfactory results are achieved with high similarities between sequences. The proposed method could enhance the compression rate without requiring extra hardware. However, some results have shown that the compression rate is relatively low with poor similarities because cluster analysis forces every sequence into a cluster despite exhibiting low correlations with other cluster members[20]. Thus, calculating the similarity of every sequence in one cluster after clustering is necessary. The data compression factor is defined as the ratio between the uncompressed and compressed sizes of sequences. Similarity is related to the compression ratio. In many cases, poor similarities have a serious effect on the compression rate. The to-be-compressed sequences could be divided into two parts in accordance with compression ratios. TRCMGene compresses the part with high similarity according to core sequences and dynamically selects special sequences and compressed sequences with poor similarity on the basis of *k*-nearest neighbours algorithm (*k*NN), a machine-learning algorithm[21]. We call this approach the two-step referential compression method (TRCM). The structure of the original data could be rebuilt without losses by using core sequences and the compressed file.

Here, we propose a two-step compression method for storing large genetic data produced by NGS. Firstly, we cluster the data using the *K*-means algorithm and preliminarily estimate the similarity of every sequence. We compress high-similarity sequences according to core sequences. Secondly, we dynamically select special sequences on the basis of *k*NN technology and compress low-similarity sequences that are highly related to the selected special sequences. We show that our method consistently performs better than the compression approach implemented in PLINK and provides an excellent compression factor for genetic data. The compressed data structure also provides the potential for the efficient implementation of permutation methods and does not require any overhead CPU time for decompression.

## Method

### Digitization of genetic data

Genetic data may be compressed efficiently by selecting for each bi-allelic marker depending on the minor allele frequency (MAF) of the respective marker[22]. Before compressed by using TRCMGene algorithm, genetic data had to be processed numerically according to the related MAF. A small fragment of genetic data was used to show the process of digitization simply, as shown in S1 Fig.

The Euclidean distance in TRCMGence was used to calculate the distance between two individuals after digitization. In a genetic file, a sequence $g_{i}{}^{e}(g_{i}^{1},g_{i}^{2},\ldots ,g_{i}^{E})$ and the collection of sequences *G* = {*g*_*i*_, *i* = 1,…, *N*}. The distance was calculated by

$$d(g_{i},g_{j})=\sqrt{{\left(g_{i}^{1}-g_{j}^{1}\right)}^{2}+{\left(g_{i}^{2}-g_{j}^{2}\right)}^{2}+\ldots +{\left(g_{i}^{E}-g_{j}^{E}\right)}^{2}}$$ (1)

Where, *d*(*g*_*i*_, *g*_*j*_) is the distance between Sequence *g*_*i*_ and Sequence *g*_*j*_.

### K-means cluster analysis for ORCM

Cluster analysis or clustering is the task of grouping a set of objects in such a manner that objects in the same group (called a cluster) are more similar to each other than to those in other groups (clusters) [23]. Clustering analysis could be used to group similar data together and show the use of the same or different distribution of fragments presented in the sequence.

The *K*-means algorithm is one of the simplest and fastest clustering algorithms[24]. All sequences of objects are represented as a point in a multidimensional feature space[25]. The *K*-means algorithm takes the number of clusters *k* as an input parameter. The program starts by randomly selecting *k* sequences as the centres of the clusters. These initial centres can be simply randomly selected from the sequences. Once some centres have been selected, the algorithm will take each sequence and calculate its distance from all cluster centres. The second step begins by considering all sequences for grouping into new clusters in accordance with the calculated distances and centre positions in new clusters. The new centre is usually obtained by calculating the mean distance of the sequences that belong to this cluster. Given that the centres have moved, the memberships need to be updated by recalculating the distance from each sequence to the new cluster centres, thereby minimising the within-cluster sum of squares [26]. The algorithm continues to update the cluster centres on the basis of the new membership. It continues to update the membership of each sequence until the cluster centres are fixed, such that no sequence moves from one cluster to another cluster[27]. Given that no sequence has changed its membership, the centres will remain the same and the algorithm will terminate. The cluster centres could be considered as the core sequences[28].

In a genetic file, a cluster *K* with centres $C_{K}^{e}(C_{K}^{1},C_{K}^{2},\ldots ,C_{K}^{E})$ and *R*_*k*_ (called radius of cluster *K*) will only contain the sequences that satisfy the following property:

$$d(g_{i},C_{K})<R_{K}$$ (2)

Eq (2) indicates that cluster *K* only contains sequences with a scale represented by *R*_*k*_. We use Euclidean distance as the distance measure in this study.

### *k*-nearest neighbor for TRCM

*k*NN is one of the oldest and most intuitive classification algorithms [29]. When paired with domain knowledge [30] or learned distance metrics[31], *k*NN is highly competitive with many machine-learning applications. With the expanding use of machine-learning algorithms in various application settings, the *k*NN rule has become particularly attractive because its predictions are easily explained.

An important drawback of *k*NN is its slow test-time performance[32]. *k*NN takes *O*(*dn*) with respect to the data dimension *d* and the training set size *n* because it must compute the distances between the test input and all elements in the training set. Similarly, the space requirements of *k*NN also include *O*(*dn*) because the entire training set has to be stored [32]. The high time and space complexity associated with *k*NN render computing the decision rule impracticable for time-critical applications and large-scale datasets—a problem that time and space is likely to remain relevant as datasets continue to grow[33]. The approach that we demonstrated in this study involves reducing the number of data by dividing the training data into subsamples in accordance with the region division method.

### Description of TRCMGene

**Fig 1:**
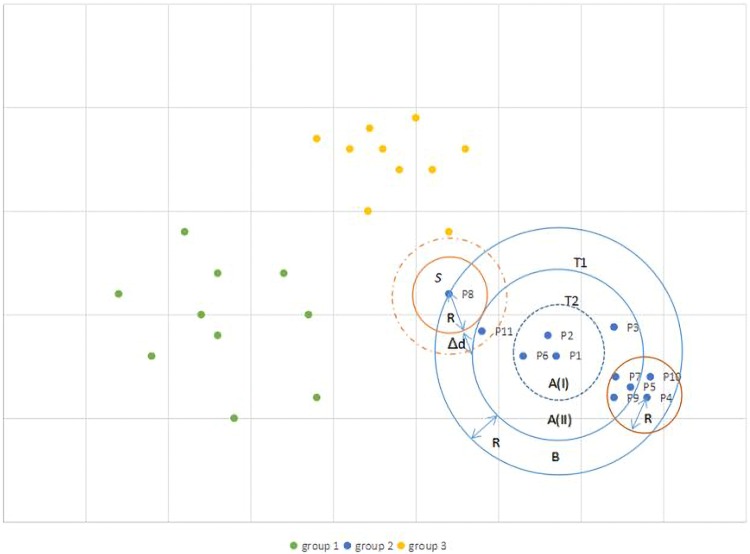
Application of TRCMGene to the toy example.

The two-step compression procedure is composed of the following steps:

1. Partition a set of sequences of objects that are being compressed into some clusters on the basis of *K*-means.
2. Calculate the distances between the cluster centre sequence and other sequences that belong to this cluster and divide the sequences into two parts in accordance with distance.
3. Directly record the index relationships of the cluster centres with the sequences that are close to the cluster centre.
4. Use *k*NN to select sequences suitable for replacing the cluster centre, as illustrated in Fig 1, and record the relevant structure for sequences that are far from the cluster centre.
5. Build a tree index structure of these sequences for sequence processing based on the data dictionary.

**Fig 2:**
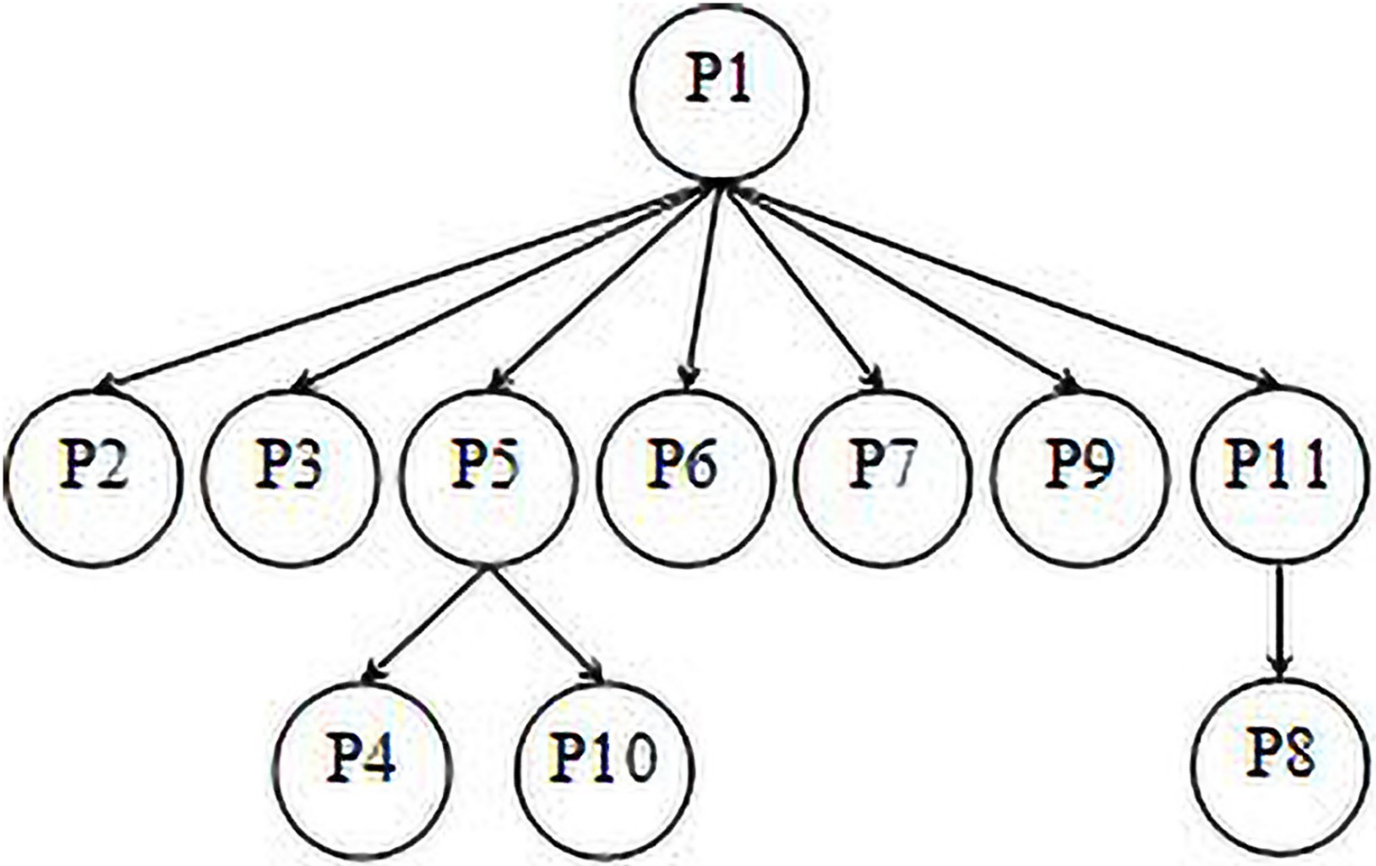
Index structure of the toy example.

Fig 1 illustrates the application of TRCMGene to a set of two-dimensional data points as a toy example. These points were divided into three groups on the basis of cluster analysis and were marked with different colours. The method used a fast approximation distance metric and two distance thresholds *T*1 > *T*2 for processing. Each group was decomposed into a circle area *A* and a ring area *B* with ring width *R* by boundary *T*1. The referential compression method could be used to directly compress each point in area *A* that could be directly compressed with cluster centre *P*1. We referred to this approach as ORCM. The distances of points in area *B* were >*T*1. Thus, for these points with low similarity to *P*1, other reference points for compression should be identified to gain high compression factor. To reduce the amount of computation and comparison, for example, area *A* was divided into *A*(*I*) and *A*(*II*) by boundary *T*2. The distances of points at area *A*(*II*) were closer to points in area *B* than those of *P*1, such that points in area *A*(*II*) could be reference points for points in area *B* in the TRCM. To some point in area *B*, we drew a circle block *S* with the centre at this point and the radius equal to *R* to decrease the number of *k*NN training sets. If one or some points at area *A*(*II*) are in Block *S*, then *k*NN would preferentially select one as reference point of this point and other points at area *B* in block *S* for compression. The relative position shown in Fig 1 indicated that the reference point of *P*4 and *P*10 is *P*5. If no points at area *A*(*II*) were in block *S*, then we gradually increased the radius of *S* by Δ*d* until some points in area *A* could be detected. The reference point of *P*8 is *P*11 in this toy example. The index structure of *P*1–*P*11 for compression, decompression and random access is shown in Fig 2.

### Index structure

TRCMGene ensures fast random access to sequences on the basis of their ID, regenerable index structure and index file. Keeping indexes enable constant time access to a given sequence or a given pattern substring. Accessing a random sequence means finding the ID in the alphabetically ordered small index file to determine its position in the compressed file. The index file was accessed directly at the ID position, which indicates the location of the sequence information that corresponds to such an ID. Finally, the compressed block containing the target ID was read and inflated in RAM, and the sequence was directly retrieved. Therefore, only a small portion of data was loaded to gain access to the sequence, and only a small portion of RAM was used even if the file size exceeded RAM size.

### Compressed file structure

**Fig 3:**
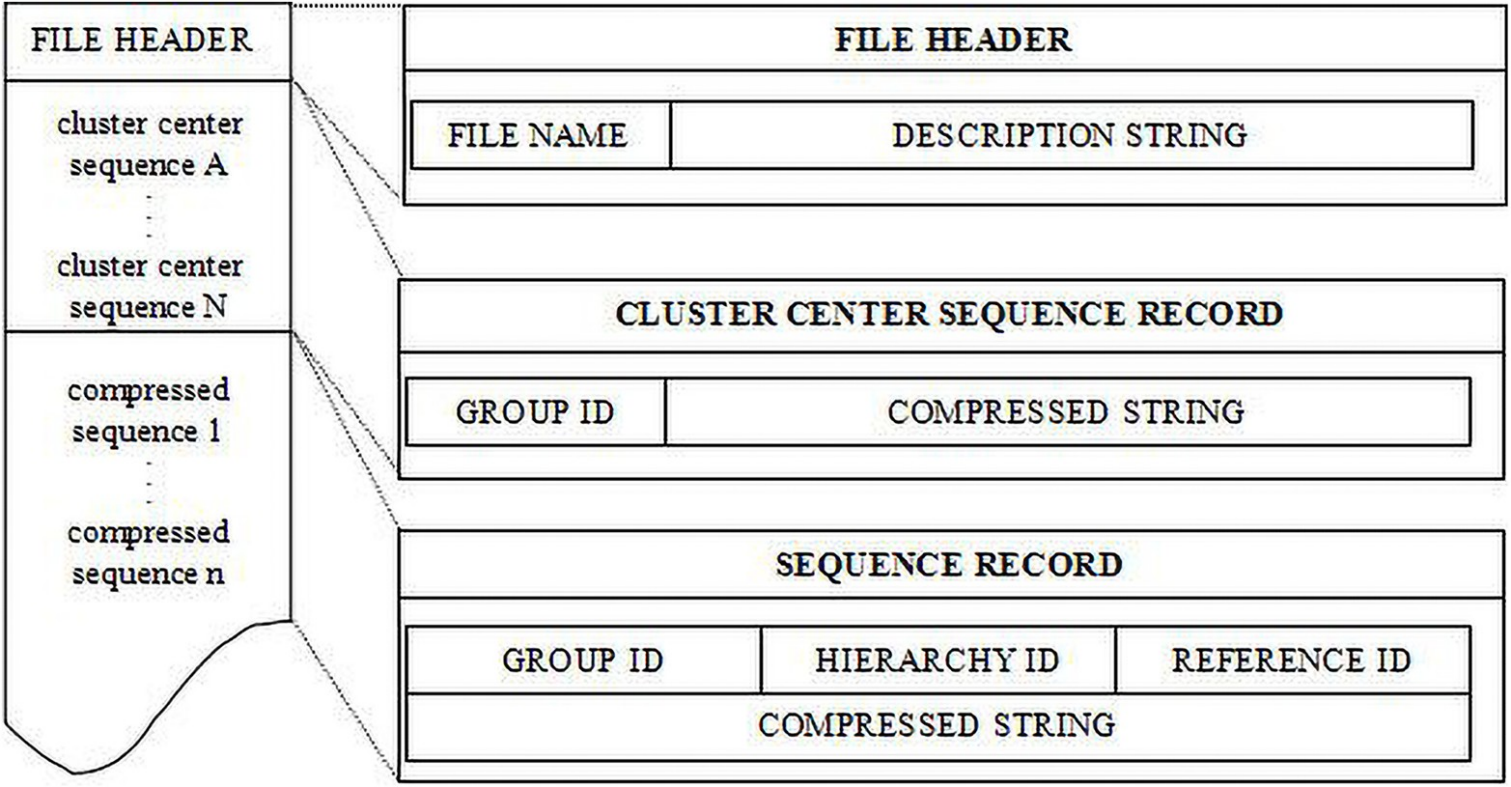
Compressed file structure. The general structure is shown on the left. The detailed description of every compressed sequence is shown on the right. Refer to the text for details.

In this study, a static data compression dictionary was adapted to enable genetic data compression after the establishment of the two-step index structure. This dictionary was created and stored in memory for use in compressing genetic data. The simplified scheme of the compression file structure of TRCMGene is shown in Fig 3. The scheme started with a *file header*, in which file details are recorded by a file name and description string with a fixed length. Then, *cluster centre sequence record* stored the compression results by using the data dictionary with replacement rule. Finally, by using the data dictionary, *sequence record* contained the group ID, hierarchy ID and reference sequence ID based on the index file and compressed results. A particular sequence could be accessed using only its compressed record instead of the entire file, thus saving time and disc usage [34]. A series of different symbols were designed for recording position information between reference and their related pre-compressed sequences. The different mode were 0, 1 and 2 for recording the differential types. Mode 0 and Mode 2 represent homozygous difference, and Mode 1 represents heterozygous difference (exemplified in S2 Fig). The scheme also served as a kind of firewall against data corruption.

## Results and discussion

### Tests

We evaluated the performance of TRCMGene on 6 real data described in Table 1. There were 4 genetic files of Maize with as the suffix, ranging from 321 MB to 100.6 GB. One of the files with 3.42G data size and another 2 species data files with “.ped” suffixes were used in Table 2. All tests were conducted on a desktop computer (Intel Core i7-3770 Quad Core Processor CPU @ 3.40 GHz and 8 GB Memory) with 64-bit Ubuntu 13.04. Time measurements were performed with the Unix **time** command.

**Table 1:** The detailed information of data files mentioned.

Species	Original File Size	The Numbers of Individuals	The Numbers of SNPs	The Numbers of Marks
Maize	321MB	115	73157	8413055
Maize	3.42G	201	459446	92348646
Maize	44.3G	702	1692698	1188273996
Maize	100.6G	1398	1928450	2695973100
Arabidopsis	4.35 GB	219	759270	166280130
Mice	611 MB	59	144782	8542138

**Table 2:** Performance of TRCMGene compared with that of two other compression methods.

Species	Original File Size	Items	TRCM Gene	PLINK	GZIP
Maize	3.42 GB	File size after compression (MB)	201.5	219.6	419.7
		Compression factor	16.97	15.57	8.15
		Compression time (s)	428	549	407
Arabidopsis	4.35 GB	File size after compression (MB)	212.2	286.1	498.3
		Compression factor	20.50	15.20	8.73
		Compression time (s)	539	719	489
Mice	611 MB	File size after compression (MB)	34.6	40.3	83.9
		Compression factor	17.68	15.16	7.28
		Compression time (s)	89	73	71

The experimental results indicated that TRCMGene is a robust development that provides an appropriate compression method for genetic data and nearly instant access to any sequence at any moment. Entire datasets could be efficiently obtained from TRCMGene.

**Fig 4:**
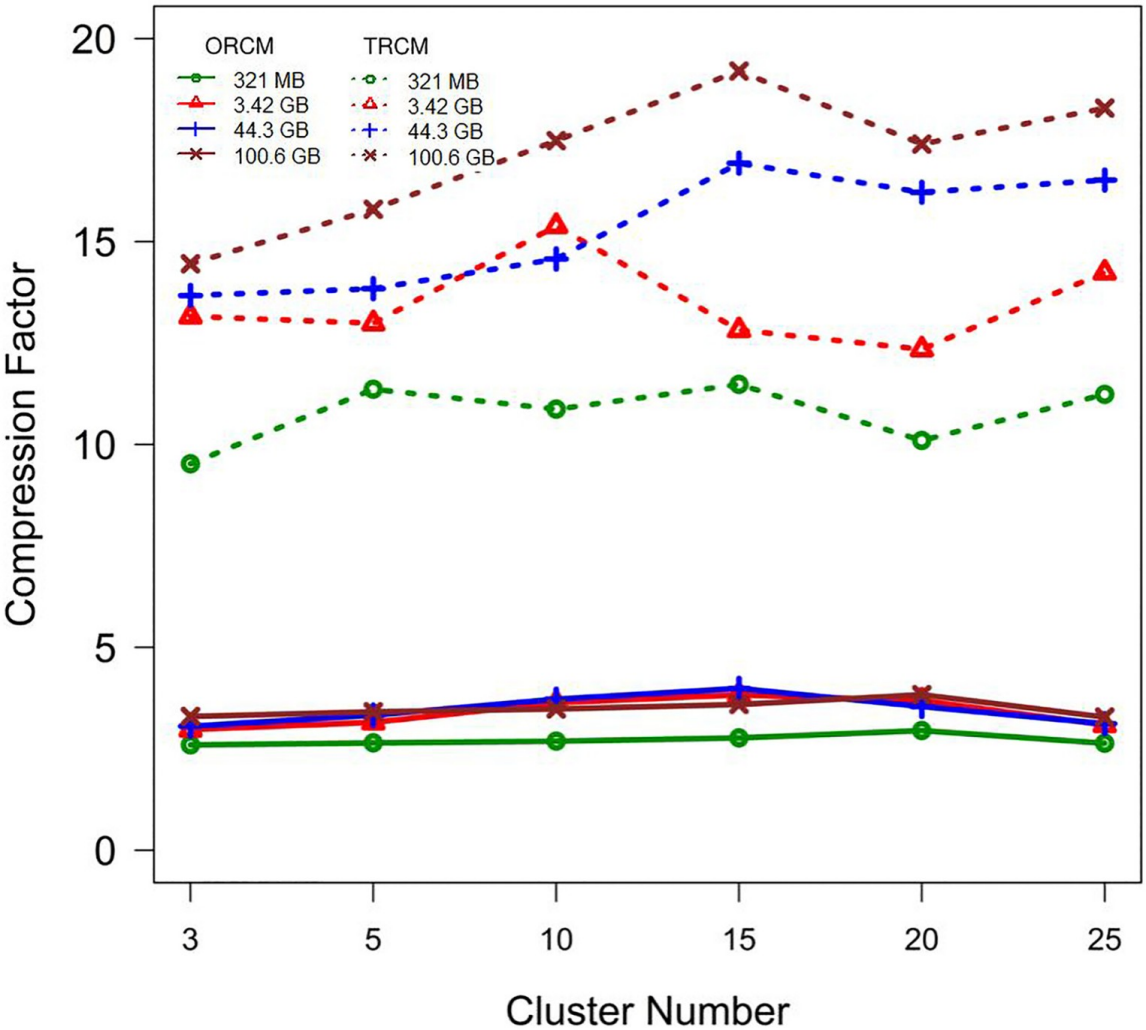
Compression capabilities of TRCMGene compared to method by ORCM. Compression factor with respect to the uncompressed file size calculated as original file sizes divided by the compressed file sizes (greater is better).

Differences between the compression factors of TRCMGene and ORCM (Fig 4) became more remarkable with file size. The compression factors of ORCM for different file sizes were nearly the same, as shown in the lower part of Fig 4. The average compression factors of TRCM were three to seven times those of ORCM. This result illustrated that the second compression process by *k*NN provides advantages.

The efficiency of TRCMGene depends on two factors: The number of clusters and the proportion of sequences compressed by TRCM. It can be seen that as the cluster number increased, compression factor of TRCMGene had a small fluctuation. The time for compressing 4 maize dataset using TRCMGene with number of clusters were shown in Table 3. The time to compress grew a little more under 15 clusters, and then it increased slightly faster. Although selecting parameter *k* was difficult in cases where external constraints were not given, parameter *k* did not have a crucial effect on compression through ORCM and TRCM. This result indicated that compression is insensitive to *k*. Thus, we usually selected a small parameter *k* to save compression time.

**Table 3:** Time needed to compress by TRCMGene.

DataSet size	Compression time (s)				
	3 clusters	5 clusters	10 clusters	15 clusters	20 clusters
321M	40	45	56	63	83
3.42G	428	449	462	488	561
44.3G	4656	4678	4689	4756	4810
100.6G	13968	14789	15239	15887	16342

**Fig 5:**
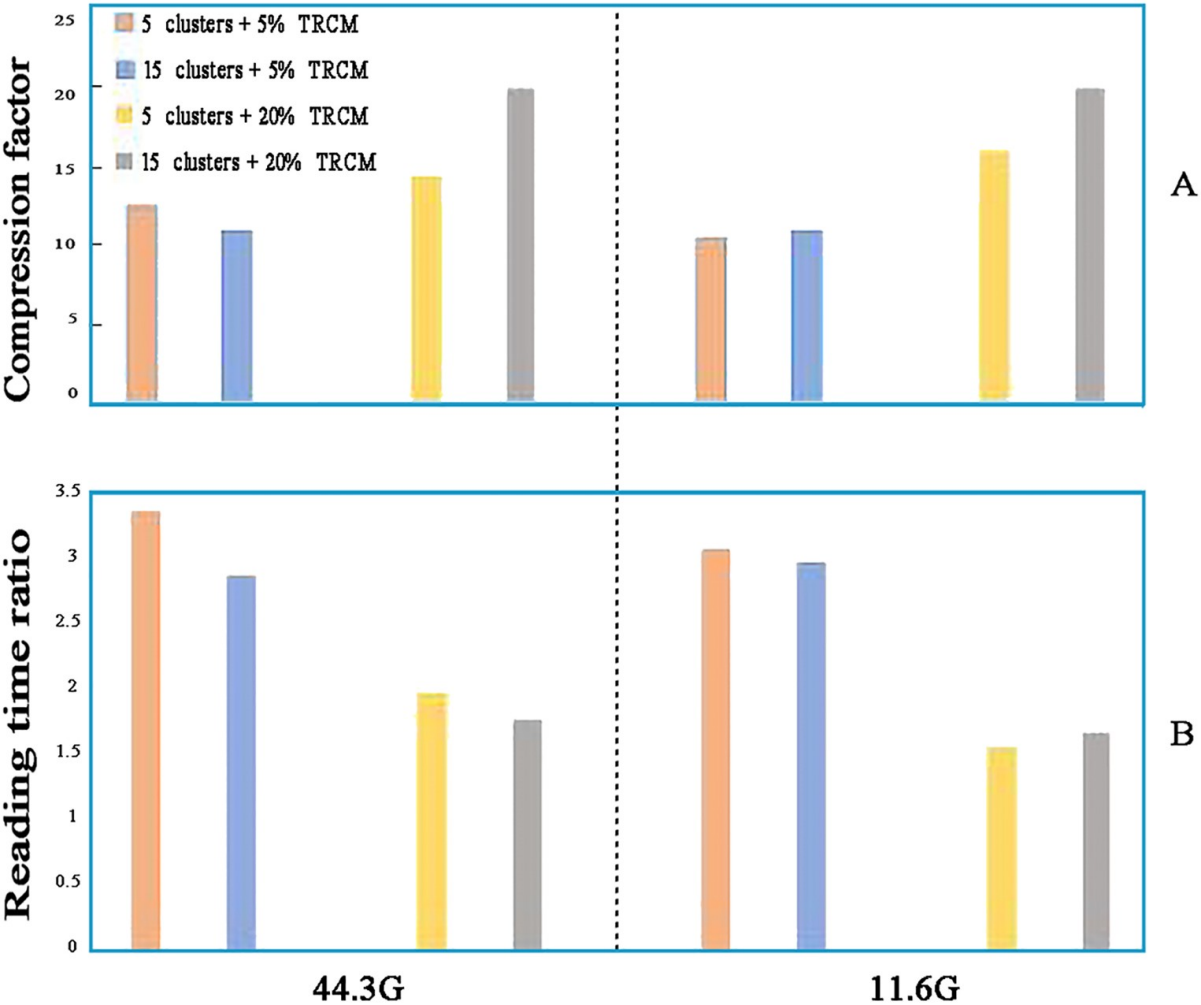
Factors that influence the performance of TRCMGene.

**Fig 6:**
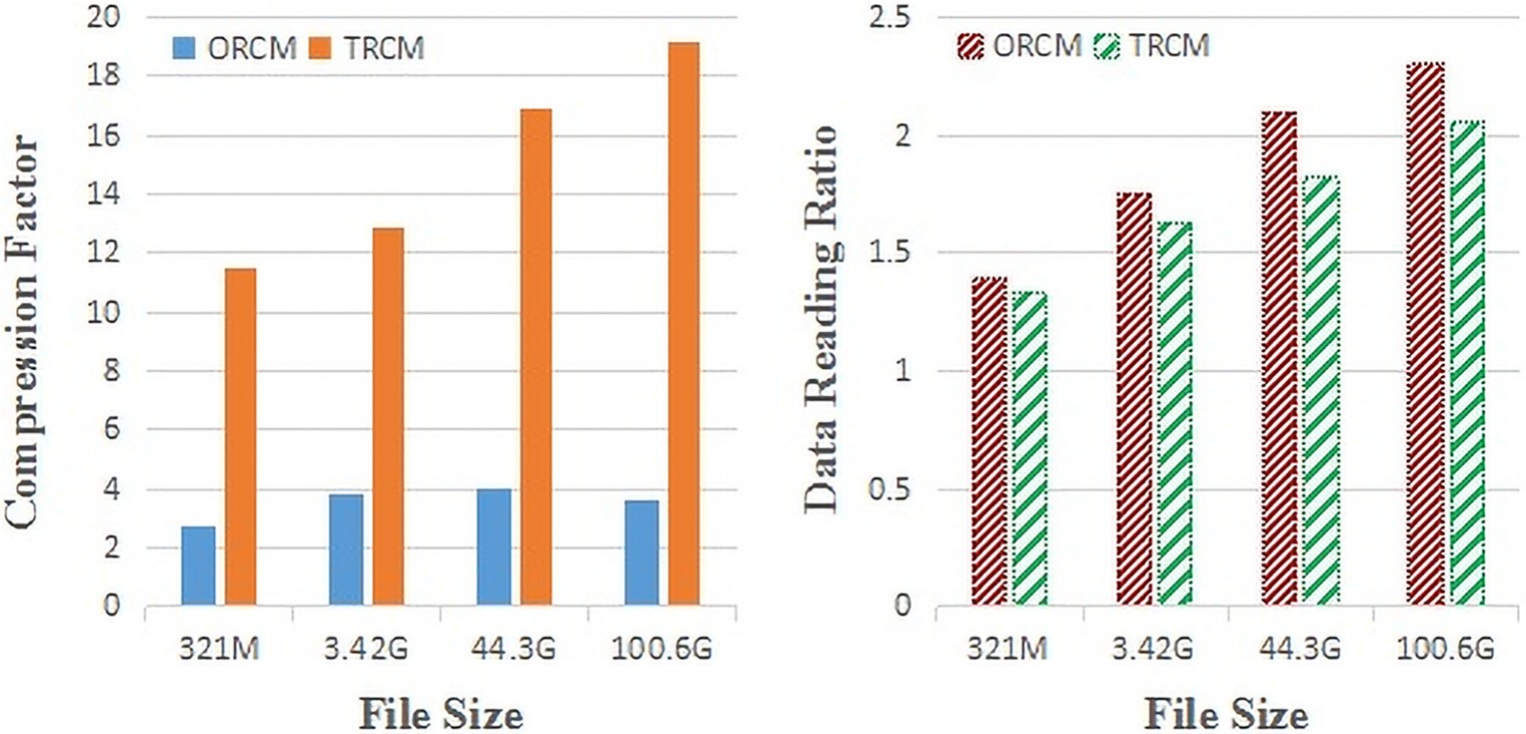
Reading capabilities of TRCMGene. Reading time ratio was defined as the ratio between the time of reading uncompressed file and the time of reading compressed file (greater is better).

Another factor that influences compression is the proportion of sequences compressed by TRCM. This proportion could improve compression performance in different numbers of clusters. Fig 5A showed the compression comparison with 5 and 15 clusters. Compressing a high proportion of sequences by TRCM would result in high compression factor but would slightly decrease the reading time. (Fig 5B). The compression factor of genetic data is limited by the complexity of the compression algorithm and compression time. Meanwhile, compression factor is related to the reading time. Fig 6 shows that TRCMGene exhibited a good balance between compression factor and reading time. The compression factors of TRCM outperformed those of ORCM in all files with different sizes, with the same trend of reading time ratio. Although compressed files have a more complex data structure than simple uncompressed original files, a reasonable index structure and data dictionary could improve the time efficiency of reading. The index structure ensured that only necessary information was loaded for the jobs, thus reducing the memory space by two thirds.

Table 2 shows the performance of TRCMGene compared with PLINK and GZip. The compression factor of TRCMGene was slightly greater than that of PLINK. TRCMGene could be used for some common genetic data formats, which outperformed PLINK only processing .ped files. GZip gave the lowest compression factor and fewest compression time of all. GZIP compared well with TRCMGene in data format. But it need decompress the compressed files before operation, thus this general-purpose compression software takes extra time to decompress the files before loading them into the memory. PLINK is designed to flexibly perform a wide range of basic, large-scale genetic analyses and could directly use compressed files for whole-genome association analysis. But compressing large files by PLINK would take much time than other two.

## Conclusion

We developed TRCMGene, a new compression method, to address the problem of large file sizes and long loading times of genetic data. We introduced a novel concept called two-step compression method, which built an index structure using *K*-means and *k*NN. We have shown that this method works better than PLINK and GZip with a good balance between compression factor and reading time. Our method utilizes the structure of the compressed data and enables the direct loading of genetic data into memory. TRCMGene not only saves disc space but also saves accessing time to the file and speeds up sequence loading. These characteristics collectively optimise the use of computer resources.

## Supporting information

S1 Fig A simple example of digitization the genetic data. If the related MAF was A/C, the allele information was coded using 0 to 2 where 0 = AA, 1 = AC and 2 = CC.
(TIF)

S2 Fig A simple example of compressed data using the static dictionary. A string “%2&0(2)1” was stored to record the difference between pre-compressed sequence and its reference sequences. When uncompressed, the pre-compressed sequence can be retrieved by reference sequence and this string.
(TIF)
