# Supplementary figures and images for: TRCMGene: A two-step referential compression method for the efficient storage of genetic data
Source: PLoS One. 2018 Nov 5;13(11):e0206521. doi: 10.1371/journal.pone.0206521 (PMC6218042; doi:10.1371/journal.pone.0206521)

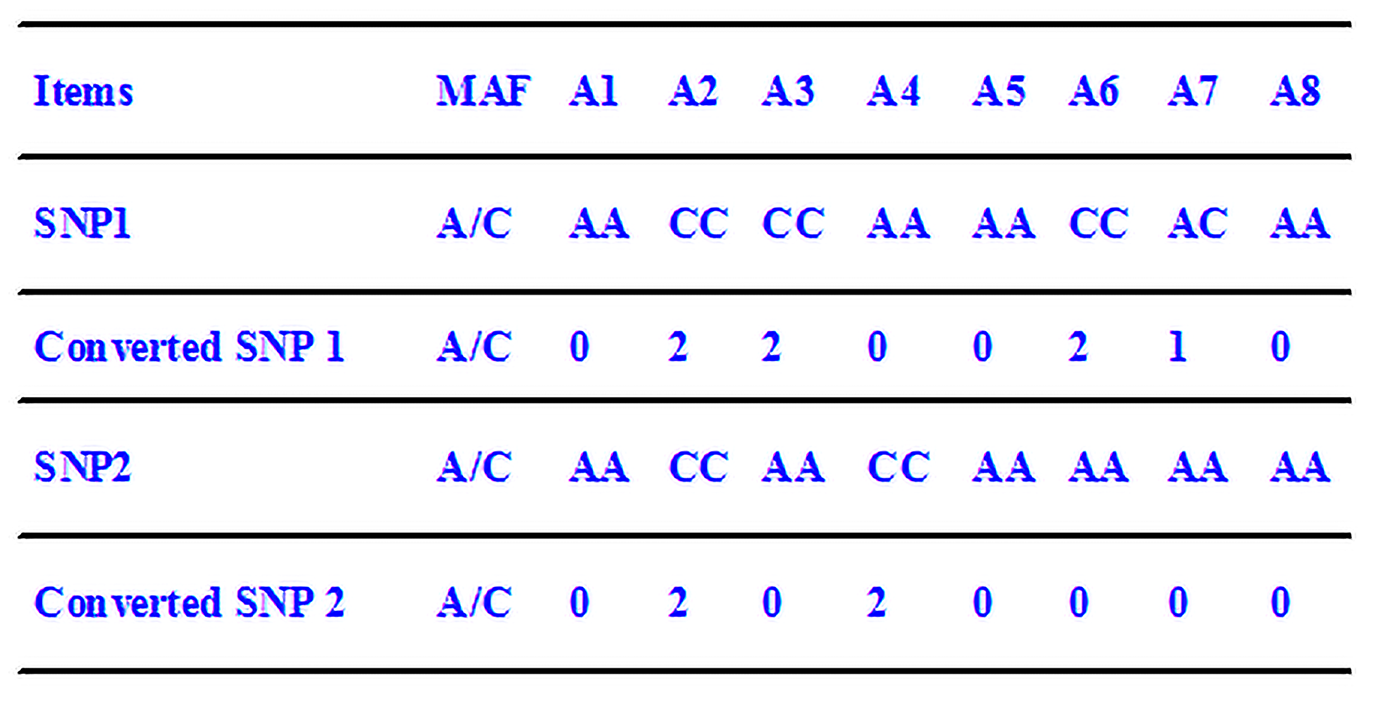

Supplement: S1 Fig — If the related MAF was A/C, the allele information was coded using 0 to 2 where 0 = AA, 1 = AC and 2 = CC. (TIF) [file pone.0206521.s001.tif]

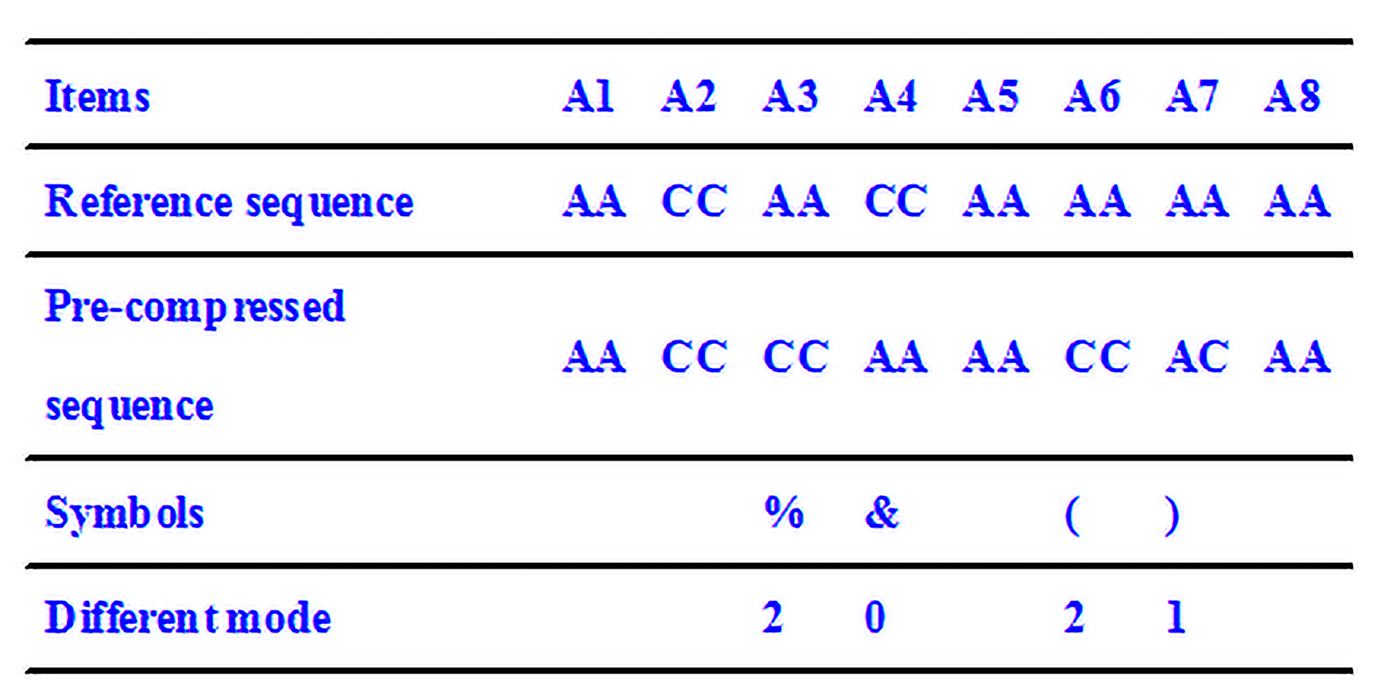

Supplement: S2 Fig — A string “%2&0(2)1” was stored to record the difference between pre-compressed sequence and its reference sequences. When uncompressed, the pre-compressed sequence can be retrieved by reference sequence and this string. (TIF) [file pone.0206521.s002.tif]
